# UBE2T regulates epithelial–mesenchymal transition through the PI3K-AKT pathway and plays a carcinogenic role in ovarian cancer

**DOI:** 10.1186/s13048-022-01034-9

**Published:** 2022-09-10

**Authors:** Ping Cui, Hao Li, Can Wang, Yuan Liu, Mengjun Zhang, Yue Yin, Zhenxing Sun, Yiru Wang, Xiuwei Chen

**Affiliations:** Department of Gynecologic Oncology, Harbin Medical University Cancer Hospital, Heilongjiang 150040 Harbin, China

**Keywords:** Epithelial–mesenchymal transition, Ubiquitin-binding enzyme E2T, Tumorigenesis, Ovarian cancer

## Abstract

**Background:**

Ubiquitin-binding enzyme E2T (UBE2T), a member of the E2 family of the ubiquitin–proteasome pathway, is associated with tumorigenesis of varioustumours; however, its role and mechanism in ovarian cancer remain unclear.

**Results:**

Our study revealed that UBE2T is highly expressed in ovarian cancer; this high expression was closely related to poor prognosis. Immunohistochemistry was used to validate the high expression of UBE2T in ovarian cancer. This is the first study to demonstrate that UBE2T expression is higher in ovarian cancer with *BRCA* mutation. Moreover, we demonstrated that UBE2T gene silencing significantly inhibited ovarian cancer cell proliferation and invasion. The epithelial–mesenchymal transition (EMT) of ovarian cancer cells and phosphatidylinositol 3 kinase/protein kinase B (PI3K-AKT) pathway were significantly inhibited. Adding the mechanistic target of rapamycin activator MHY1485 activated the PI3K-AKT pathway and significantly restored the proliferative and invasive ability of ovarian cancer cells. Furthermore, a tumorigenesis experiment in nude mice revealed that tumour growth on mice body surface and tumour tissue EMT were significantly inhibited after UBE2T gene silencing.

**Conclusions:**

This study demonstrated that UBE2T regulates EMT via the PI3K-AKT pathway and plays a carcinogenic role in ovarian cancer. Moreover, UBE2T may interact with *BRCA* to affect ovarian cancer occurrence and development. Hence, UBE2T may be a valuable novel biomarker for the early diagnosis and prognosis and treatment of ovarian cancer. Further, UBE2T inhibition may be effective for treating ovarian cancer.

**Supplementary Information:**

The online version contains supplementary material available at 10.1186/s13048-022-01034-9.

## Background

Ovarian cancer ranks first among gynaecological malignant tumours in terms of morbidity and mortality [1]. Its current standard treatment is platinum-based chemotherapy and surgery. Despite advances in surgical techniques, chemotherapy, targeted therapy and immunotherapy, the therapeutic outcome of this disease remains unsatisfactory. Although the majority of patients initially respond to treatment, most will relapse and require chemotherapy for their entire survival period [2]. Of note, the 5-year survival rate among such patients is only 47% [3–5]. The poor prognosis of ovarian cancer is directly related to the lack of typical clinical symptoms and specific sensitive markers in the early stage of the disease. Also, ovarian cancer affects the quality of life of patients. It is also related to many mechanisms, such as the angiogenic effects in ovarian cancer and mesothelial cells induced by Cu [6], high expression of Tsen CD8 T cells in ascites [7] and invasion of malignant ascites-derived EVs [8]. Hence, there is an urgent need to study the pathogenesis of this disease and discover sensitive and efficient markers for its effective diagnosis and treatment.

At present, the occurrence of ovarian cancer is closely related to the phosphatidylinositol 3 kinase-protein kinase B-mechanistic target of rapamycin (PI3K-AKT-MTOR) pathway. The mechanistic target of the PI3K-AKT-MTOR pathway (PI3K pathway) is the most common change signal, which exists in 70% of patients with ovarian cancer [9, 10]. The activation of the PI3K pathway is related to the invasive phenotype, chemotherapy resistance and poor prognosis of ovarian cancer [6], rendering it an important target for treatment. However, despite the high frequency of activation of the PI3K pathway in ovarian cancer, the clinical activity of inhibitors of this pathway is limited thus far [11]. Therefore, the development of new therapeutic targets is necessary to improve the inhibition of the PI3K pathway.

Epithelial–mesenchymal transition (EMT) is an important process in the transformation of normal cells to cancer cells; it has been reported that EMT promotes various types of cancer [12]. This is a reversible developmental process used by cancer cells to reversibly switch from an epithelial phenotype with tip-base polarity and cell–cell adhesion to a more motor stromal state with spindle shape and front-back polarity [13]. In this process, E-cadherin (an important component of adhesion junction), as well as atresia proteins, claudin, epithelial cell adhesion molecule, α6β4 integrin and different cytokeratins (which play an important role in stabilising desmosomes) were inhibited. Moreover, the expression of vimentin, fibronectin, neurocadherin (N-cadherin), β1 and β3 integrin and matrix metalloproteinase was upregulated [14].

Ubiquitin plays a role in protein–protein interactions, localisation and enzyme activity. Also, it affects cellular processes, including transcription, DNA damage signals and DNA repair, cell cycle progression, endocytosis, apoptosis, etc. [15]. Post-translational modification, which regulates protein ubiquitination and stability through the ubiquitin–proteasome system, is considered a key regulator of cell proliferation, invasion, differentiation and death [16, 17].

Ubiquitin-binding enzyme E2T (UBE2T) is a member of the E2 family in the ubiquitin–proteasome pathway, which plays a key role in cell cycle progression, signal transduction and tumorigenesis [18, 19]. It has been associated with tumorigenesis and the development of various tumours. Some studies have found that the overexpression of UBE2T may promote the occurrence of breast cancer during the whole process of BRCA1 downregulation [20]. UBE2T is highly expressed in bladder cancer, and its deletion significantly inhibits the proliferation and colony-forming ability of bladder cancer cells [21]. It is also upregulated in hepatocellular carcinoma and plays a carcinogenic role via p53 ubiquitination [22, 23].

In addition, a number of studies have confirmed that UBE2T plays a carcinogenic role in a variety of cancers, including lung cancer, gastric cancer, renal cell carcinoma, osteosarcoma, prostate cancer and glioma [24–29]. However, the expression levels, mechanism and clinical significance of UBE2T in ovarian cancer are unclear. In this study, we analysed the expression of UBE2T in ovarian cancer and analysed the specific mechanism through which it regulates cell proliferation. These findings demonstrate the role of UBE2T in the development of ovarian cancer and may provide a new treatment strategy for this disease.

## Results

### Abnormal expression of UBE2T in ovarian cancer and its prognostic significance

**Fig. 1:**
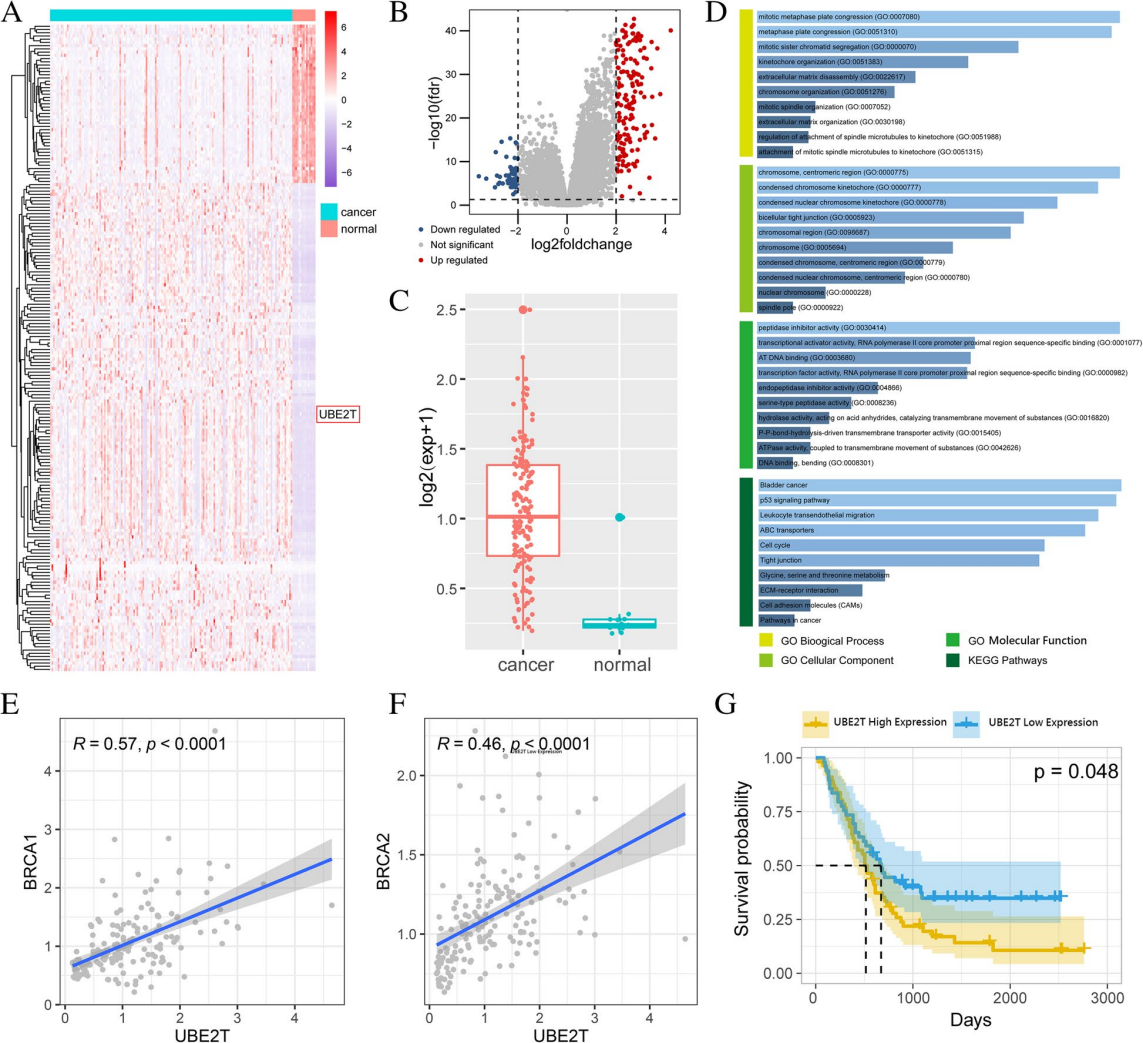
**A** Heatmap constructed using the differential genes between cancer tissues and normal tissues detected using significance analysis in the GSE51088 database. **B** Differential expression of genes in ovarian cancer according to the GSE51088 dataset. fdr, false discovery rate; UBE2T, ubiquitin-binding enzyme E2T. **C** The *UBE2T* gene was significantly upregulated in ovarian cancer samples. UBE2T, ubiquitin-binding enzyme E2T. **D** Functional enrichment of the *UBE2T* gene according to GO and KEGG analyses. ABC, ATP-binding cassette; AT, adenine–thymine; ECM, extracellular matrix; GO, Gene Ontology; KEGG, Kyoto Encyclopedia of Genes and Genomes; UBE2T, ubiquitin-binding enzyme E2T. **E** Spearman rank correlation analysis of *UBE2T* and breast cancer gene *BRCA1*. UBE2T, ubiquitin-binding enzyme E2T. **F** Spearman rank correlation analysis of *UBE2T* and breast cancer gene *BRCA2*. UBE2T, ubiquitin-binding enzyme E2T. **G** Survival analysis of all epithelial ovarian cancer samples in the GSE51088 dataset

In this study, we first analysed the differential expression of genes in ovarian cancer using the GSE51088 dataset. The analysis revealed 200 differentially expressed genes (151 upregulated and 49 downregulated) (Fig. 1A–B). We noticed that the gene UBE2T was significantly upregulated in ovarian cancer tissues (Fig. 1C).

Subsequently, we carried out Spearman rank correlation analysis of UBE2T and breast cancer genes (BRCA1, BRCA2). The results showed a significant correlation between UBE2T and the expression of these genes in ovarian cancer (Fig. 1E–F).

We sought to further investigate the functions that may be activated by UBE2T. For this purpose, we used data from the GO database to examine the functional enrichment of the UBE2T gene by GO and KEGG analyses. The most significant biological processes of GO include extracellular matrix decomposition, extracellular matrix synthesis, two-cell tight junction, cell adhesion molecules, tumour pathway, etc. (Fig. 1D).

High Group indicates the low expression group of UBE2T with good prognosis, and Low Group represents the group with high expression of UBE2T with poor prognosis. The survival analysis of all epithelial ovarian cancer samples included in the GSE51088 dataset demonstrated that the high expression of the UBE2T gene was associated with poor prognosis (Fig. 1G).

### UBE2T expression in tissues and cell lines

#### Expression of UBE2T in tissues

**Fig. 2:**
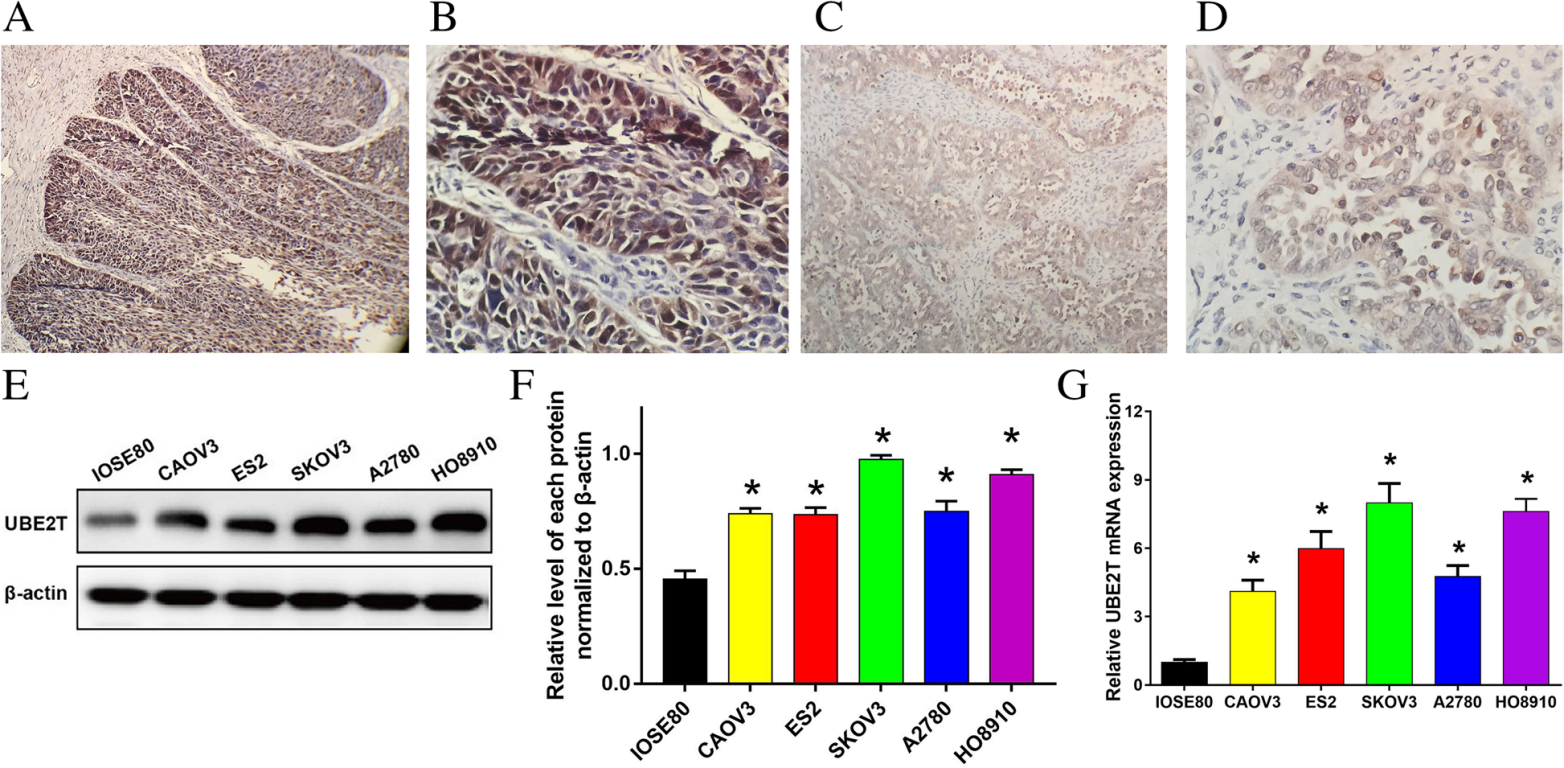
**A** High expression of UBE2T (low-power field) in ovarian serous adenocarcinoma. UBE2T, ubiquitin-binding enzyme E2T. **B** High expression of UBE2T (high-power field) in ovarian serous adenocarcinoma. UBE2T, ubiquitin-binding enzyme E2T. **C** Low expression of UBE2T (low-power field) in ovarian serous adenocarcinoma. UBE2T, ubiquitin-binding enzyme E2T. **D** Low expression of UBE2T (high-power field) in ovarian serous adenocarcinoma. UBE2T, ubiquitin-binding enzyme E2T. **E** and **F** Protein expression of UBE2T in ovarian cancer cell lines. UBE2T, ubiquitin-binding enzyme E2T. **G** Expression of UBE2T mRNA in ovarian cancer cell lines. UBE2T, ubiquitin-binding enzyme E2T

We investigated the expression of UBE2T in ovarian cancer and normal ovarian tissues by immunohistochemistry. We observed that UBE2T was expressed in the cytoplasm and nucleus (Fig. 2A–D). The histological subtype of this image is ovarian serous cystadenocarcinoma.

UBE2T was highly expressed in ovarian cancer tissues compared with normal ovarian tissues (54.3% and 25.5%, respectively); this difference was statistically significant (Table 1).

**Table 1:** Positive expression of UBE2T in ovarian cancer and normal ovarian tissues

Group	Number	UBE2T			χ^2^	*P*
		Positive	Negative	Positivity rate (%)		
Ovarian cancer tissue	70	38	32	54.3	13.528	0.000
Normal ovarian tissue	55	12	43	21.8		

*UBE2T* Ubiquitin-binding enzyme E2T

Moreover, we investigated the difference in UBE2T expression in ovarian cancer tissues with different BRCA mutations. The findings demonstrated that UBE2T was highly expressed in ovarian cancer tissues with BRCA mut versus those with BRCA wnt (74.3% and 34.3%, respectively); this difference was statistically significant (Table 2). Also, a significant positive correlation between the *UBE2T* and *BRCA* genes was noted. UBE2T expression was higher in ovarian cancer tissue with BRCA mutations. Thus, these genes may be cooperatively involved in the mechanisms underlying the occurrence and development of ovarian cancer.

**Table 2:** Positive expression of UBE2T in ovarian cancer with or without *BRCA* mutations

Group	Number	UBE2T			χ^2^	*P*
		Positive	Negative	Positivity rate (%)		
Ovarian cancer tissues with BRCA ( +)	35	26	9	74.3	11.283	0.001
Ovarian cancer tissues with BRCA ( −)	35	12	23	34.3		

*UBE2T* Ubiquitin-binding enzyme E2T

#### Expression of UBE2T in cell lines

We studied the protein expression of UBE2T in ovarian cancer cell lines using western blotting. The results showed that the expression of UBE2T protein in five ovarian cancer cell lines (i.e. CAOV3, SKOV3, ES2, HO8910 and A2780) was significantly higher than that measured in a normal ovarian epithelial cell line (IOSE80). Among them, the expression of UBE2T protein was higher in SKOV3 and HO8910 cells (Fig. 2E–F).

RT-PCR assay was used to analyse the expression levels of UBE2T mRNA in ovarian cancer cell lines. The results showed that the mRNA expression levels of the UBE2T gene in the five ovarian cancer cell lines (i.e. CAOV3, SKOV3, ES2, HO8910 and A2780) were significantly higher than those recorded in the normal ovarian epithelial cell line (IOSE80). Among them, the expression levels of UBE2T mRNA were higher in SKOV3 and HO8910 cells (Fig. 2G).

#### Silencing UBE2T

**Fig. 3:**
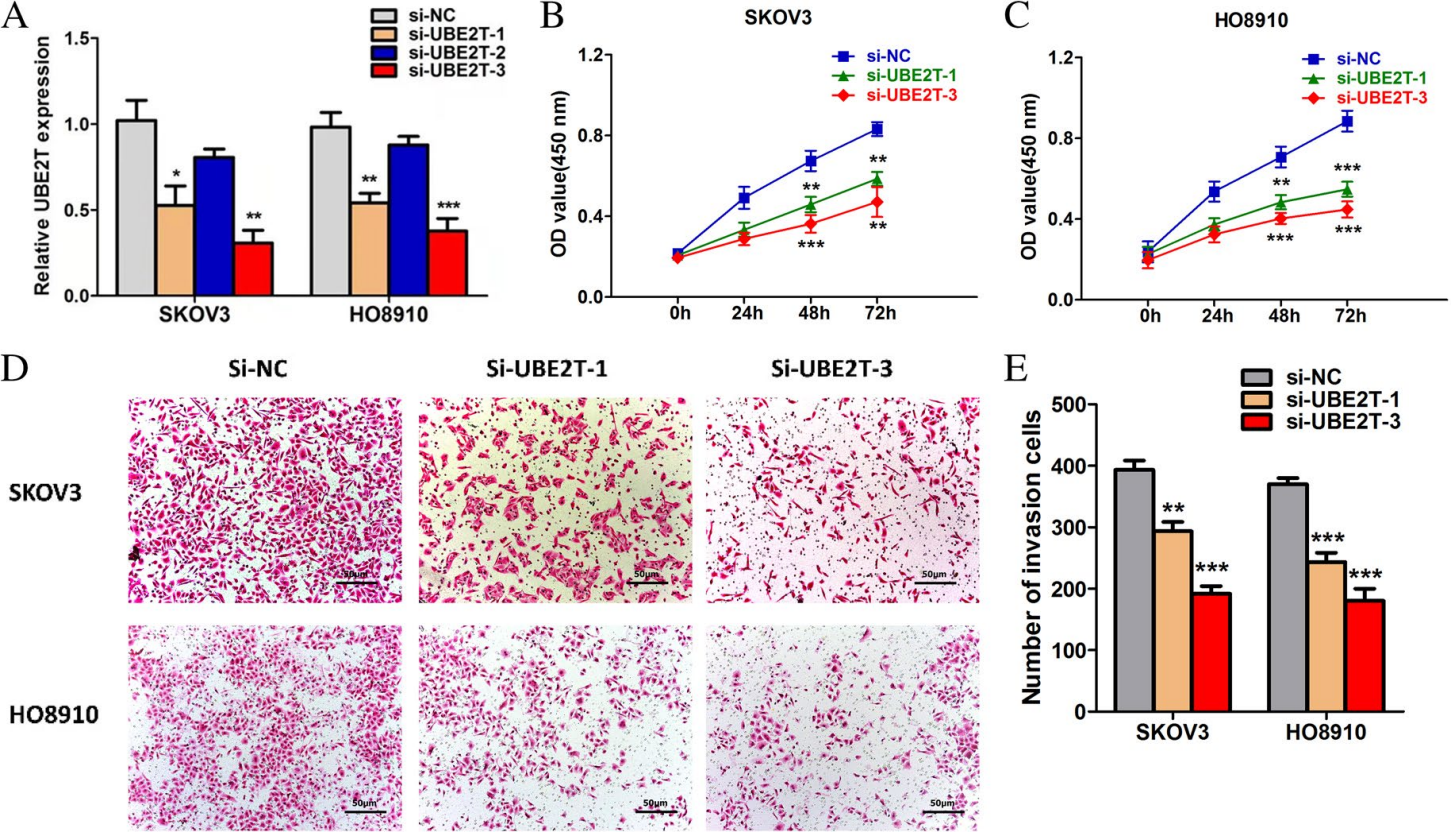
**A** UBE2T expression was efficiently knocked down by siRNAs in SKOV3 and HO8910 cells. siRNA, small interfering RNA; UBE2T, ubiquitin-binding enzyme E2T. **B ***UBE2T* knockout significantly inhibited ovarian cancer cell proliferation (SKOV3). OD, optical density; si-NC, small interfering RNA-negative control; UBE2T, ubiquitin-binding enzyme E2T. **C ***UBE2T* knockout significantly inhibited ovarian cancer cell proliferation (HO8910). OD, optical density; si-NC, small interfering RNA-negative control; UBE2T, ubiquitin-binding enzyme E2T. **D ***UBE2T* knockout significantly inhibited the invasive ability of ovarian cancer cells. si-NC, small interfering RNA-negative control; UBE2T, ubiquitin-binding enzyme E2T. **E ***UBE2T* silencing significantly decreased the invasive ability of ovarian cancer cells. si-NC, small interfering RNA-negative control; UBE2T, ubiquitin-binding enzyme E2T

We designed three siRNAs that can silence UBE2T and transfected them into ovarian cancer cell lines SKOV3 and HO8910. Subsequently, we used empty vector as NC and performed RT-PCR to test the silencing effect of the three siRNA on UBE2T in SKOV3 and HO8910 cells. As shown in Fig. 3A, si-UBE2T-1 and si-UBE2T-3 showed good silencing effect in both SKOV3 and HO8910 cell lines. Therefore, to reduce the risk of deviation from the target effect, we selected si-UBE2T-1 and si-UBE2T-3 for the subsequent experiments.

#### Silencing of UBE2T inhibited the proliferation of ovarian cancer cells

The MTT assay was used to determine the role of UBE2T in the proliferation of SKOV3 and HO8910 cells. The experimental results revealed that the proliferative ability of SKOV3 and HO8910 cells was significantly weakened after silencing of the UBE2T gene compared with the control group (Fig. 3B–C). This suggested that UBE2T promotes the proliferation of ovarian cancer cells.

#### Silencing of UBE2T significantly decreased the invasive ability of ovarian cancer cells

The Transwell assay showed that the invasive ability of ovarian cancer cells (SKOV3 and HO8910) was significantly decreased after UBE2T silencing (Fig. 3D–E). The results demonstrated that UBE2T promotes the invasion of ovarian cancer cells, and *UBE2T* knockout significantly inhibits the invasion of these cells.

### UBE2T regulated the invasive ability of ovarian cancer cells by regulating EMT of ovarian cancer cells through the PI3K-AKT pathway

#### UBE2T may act on the PI3K-AKT pathway through MTOR targets and promote the phosphorylation of PI3K and AKT

**Fig. 4:**
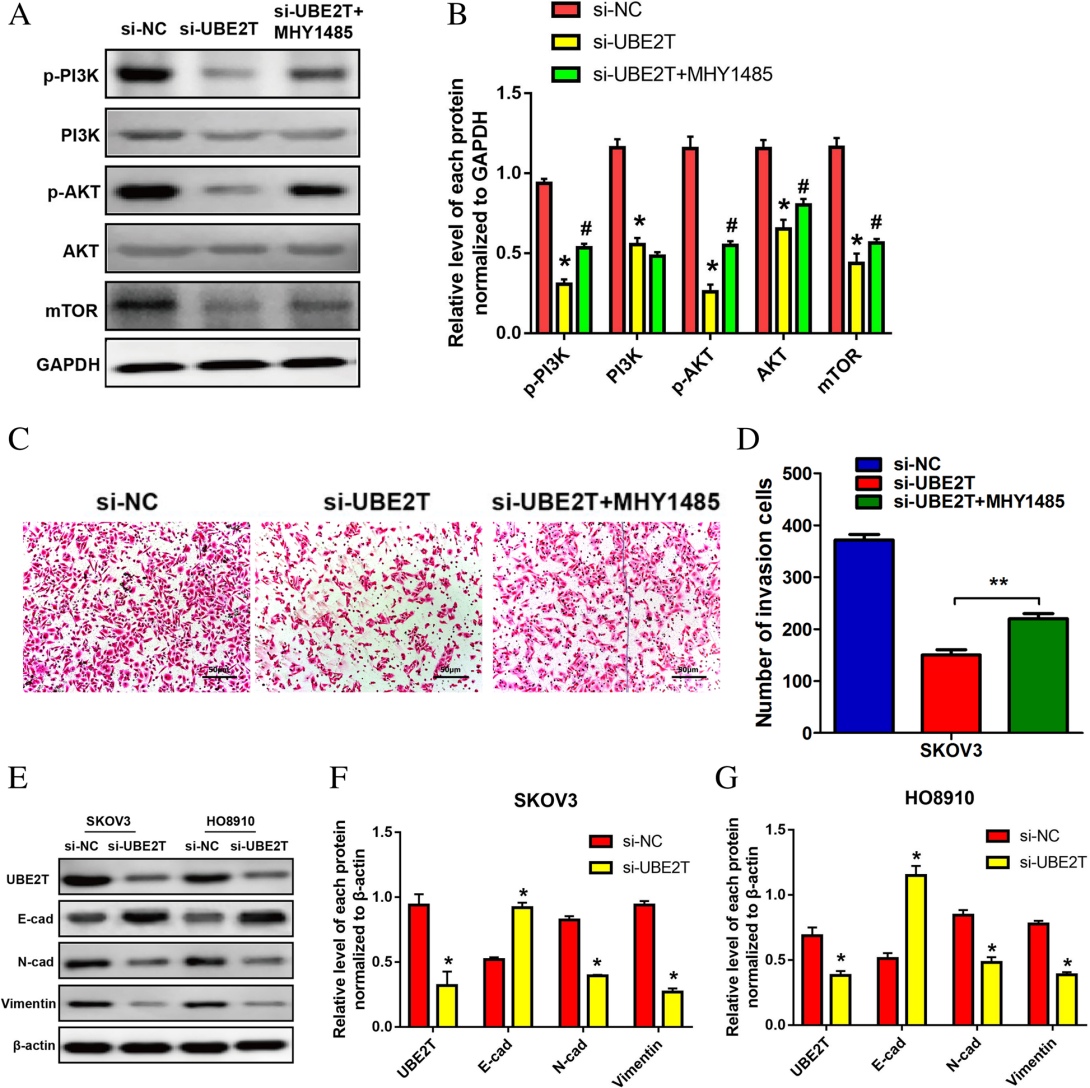
**A** and **B** Expression of key proteins in the PI3K-AKT signaling pathway after silencing of UBE2T and addition of MHY1485. (*P < 0.05 versus si-NC; #*P* < 0.05 versus si-UBE2T). Data are presented as mean ± standard deviation and analyzed via repeated-measures analysis of variance (ANOVA). The experiment was repeated thrice, independently. GAPDH, glyceraldehyde-3-phosphate dehydrogenase; MTOR, mechanistic target of rapamycin; PI3K-AKT, phosphatidylinositol 3 kinase/protein kinase B; p-PI3K, phosphorylated-PI3K; si-NC, small interfering RNA-negative control; UBE2T, ubiquitin-binding enzyme E2T. **C** and **D** Changes in the invasive ability of SKOV3 cells after silencing of UBE2T and addition of MHY1485. si-NC, small interfering RNA-negative control; UBE2T, ubiquitin-binding enzyme E2T. **E**–**G** Expression of key proteins in EMT after silencing of UBE2T in SKOV3 and HO8910 cells. *P < 0.05. Data are presented as mean ± standard deviation and analyzed via repeated-measures analysis of variance (ANOVA). The experiment was repeated thrice, independently. EMT, epithelial–mesenchymal transition; E-cad, E-cadherin; N-cad, neurocadherin; si-NC, small interfering RNA-negative control; UBE2T, ubiquitin-binding enzyme E2T

We hypothesised that UBE2T regulates the invasive ability of ovarian cancer cells by regulating their EMT via the PI3K-AKT pathway. We measured the protein expression levels of the core components of the PI3K-AKT pathway in SKOV3 cells. As shown in Fig. 4A–B, after UBE2T silencing, the expression of p-PI3K, PI3K, p-AKT, AKT and mTOR (key proteins of the PI3K-AKT pathway) was significantly decreased. We used 1 μM/ml MHY1485 (Celek, Houston, TX, USA) to activate mTOR in the PI3K-AKT signal pathway in cells in which UBE2T was silenced. As shown in Fig. 4A–B, the expression levels of the mTOR protein in the PI3K-AKT pathway increased, and those of p-PI3K, p-AKT and AKT increased significantly. In contrast, the expression levels of PI3K protein did not increase significantly. These findings indicated that UBE2T acts on the PI3K-AKT pathway via mTOR targets and promotes the phosphorylation of PI3K and AKT.

#### UBE2T promoted the proliferation and invasion of ovarian cancer cells through the PI3K-AKT pathway

Following the inhibition of UBE2T, the invasive ability of cells was significantly inhibited. Subsequently, these cells were treated with MHY1485 (an activator of the PI3K-AKT pathway). The results showed that the activator of the PI3K-AKT pathway can reverse the inhibition of SKOV3 cell invasion after UBE2T silencing (Fig. 4C–D). The findings also suggested that the depletion of UBE2T inhibits the invasion of ovarian cancer cells via inhibition of the PI3K-AKT pathway. Collectively, these findings indicated that UBE2T promotes the proliferation and invasion of ovarian cancer cells via the PI3K-AKT pathway.

#### UBE2T affected the function of ovarian cancer cells by regulating EMT

Compared with the control group, following inhibition of UBE2T in SKOV3 and HO8910 cells, the expression of E-cadherin was increased. In contrast, the expression of N-cadherin and vimentin was significantly decreased (Fig. 4E–G). These results indicated that EMT of ovarian cancer SKOV3 and HO8910 cells was significantly inhibited after UBE2T silencing. This suggests that UBE2T can affect ovarian cancer cell function by regulating EMT occurrence.

### *In vivo* xenograft tumour models confirmed that silencing of UBE2T inhibited the growth of transplanted tumours

**Fig. 5:**
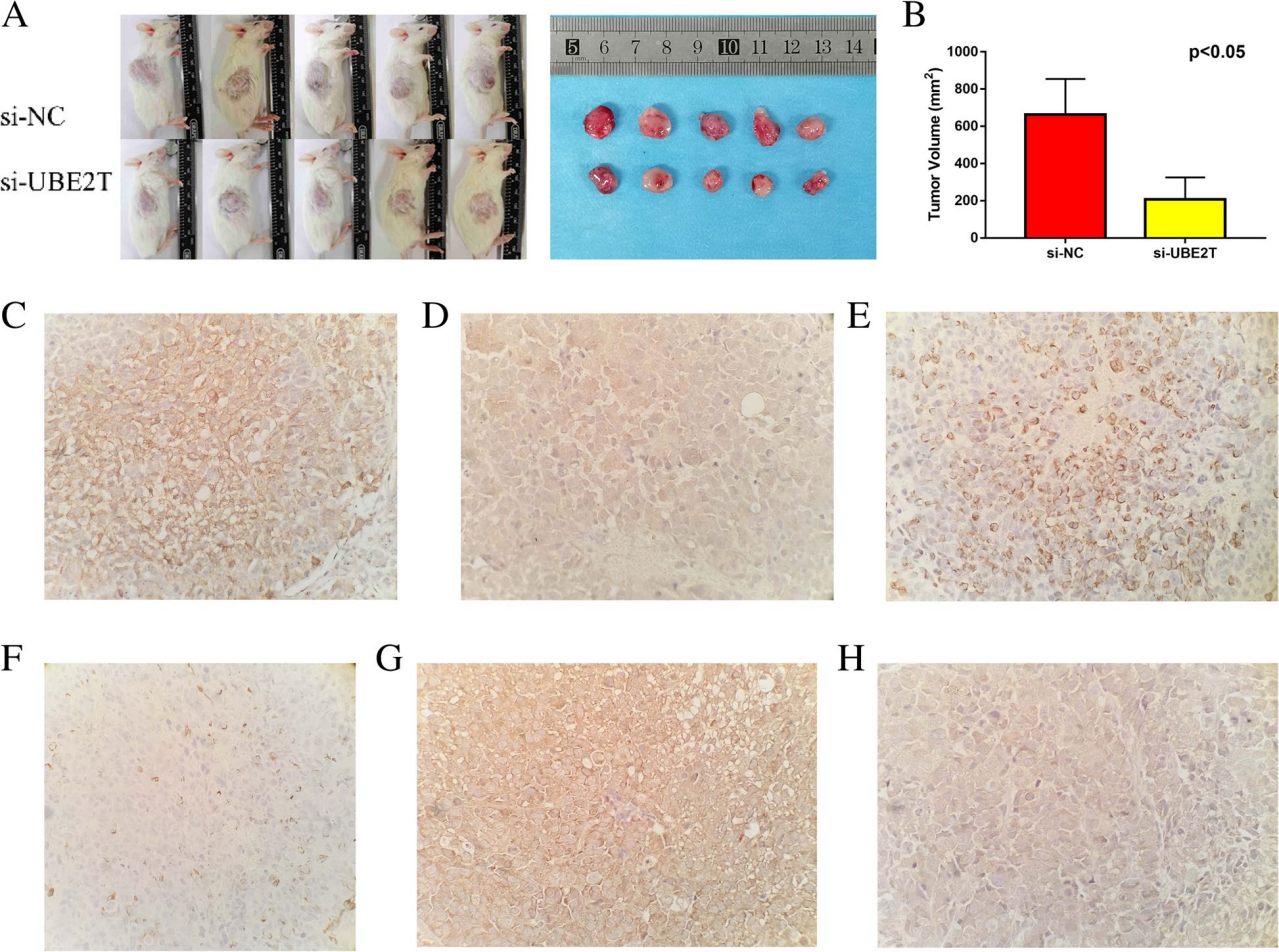
**A** SKOV3 cells transfected with si-NC or si-UBE2T were subcutaneously injected into the axilla of the right forelimb of SCID mice. The volume of each tumor was measured at a specified time. SCID, severe combined immunodeficiency; si-NC, small interfering RNA-negative control; UBE2T, ubiquitin-binding enzyme E2T. **B** The average tumor volume in mice treated with si-UBE2T was significantly lower than that in the control group. UBE2T, ubiquitin-binding enzyme E2T. **C** High expression of E-cad. **D** Low expression of E-cad. **E** High expression of vimentin. **F** Low expression of vimentin. **G** High expression of N-cad. **H** Low expression of N-cad. E-cad, E-cadherin; N-cad, neurocadherin

Three weeks after tumour formation, there was no significant difference in body weight between the si-NC and si-UBE2T groups. However, the growth of transplanted tumours was significantly inhibited after knockout of the UBE2T gene (Fig. 5A). The average tumour volume in mice treated with si-UBE2T was significantly lower than that measured in the control group (49.69 mm^3^ vs. 18.23 mm^3^, respectively; *P* < 0.05) (Fig. 5B). Therefore, we believe that UBE2T promotes the growth of transplanted tumours *in vivo*.

Immunohistochemical analysis revealed that the rate of positive expression of E-cadherin (Fig. 5C–D) was 60% and 100% in the si-NC and si-UBE2T groups, respectively. This indicated that the expression of E-cadherin was significantly increased in the latter group. The rate of positive expression of vimentin (Fig. 5E–F) was 80% and 20% in the si-NC and si-UBE2T UBE2T groups, respectively. These results indicated that the expression of vimentin was significantly decreased in the UBE2T group. The rate of positive expression of N-cadherin (Fig. 5G–H) was 60% and 20% in the si-NC and si-UBE2T groups, respectively. These findings indicated that the expression of N-cadherin was significantly decreased in the UBE2T group. This shows that UBE2T silencing inhibits EMT of ovarian cancer cells. In other words, *in vivo* experiments in mice demonstrated that UBE2T promotes EMT in ovarian cancer cells.

## Discussion

Approximately 80% of patients with ovarian cancer repeatedly relapse within 5 years after treatment, which has an adverse impact on their quality of life [4]. Repeated chemotherapy and the development of drug resistance are the main causes of poor quality of life and prognosis. Therefore, the main target for improving the prognosis is the inhibition of tumour growth and prevention of resistance to anti-tumour drugs. UBE2T is a member of the ubiquitin-binding enzyme family. Numerous studies have shown that UBE2T, as an oncogene, plays a role in a variety of tumours, including breast cancer, liver cancer, lung cancer, prostate cancer, kidney cancer, bladder cancer, glioma, etc. [20, 22, 25, 27, 29–32]. However, its molecular mechanism differs between the various types of tumours. In hepatocellular carcinoma cells, Liu et al. [18] found that UBE2T regulates the G2/M transition through the cyclin B1-CDK1 pathway and promotes proliferation and invasion. Another study showed that UBE2T promotes the occurrence of hepatocellular carcinoma by promoting the ubiquitination and degradation of p53 [22]. It was also found that silencing of UBE2T in bladder or gastric cancer could induce cell cycle arrest in the G2/M phase, thus promoting cell apoptosis and inhibiting tumour growth [21, 26]. In addition, some studies have shown a downregulation of UBE2T in renal cell carcinoma [27] and osteosarcoma [28] cells. This effect reduces the activity of the PI3K/AKT signalling pathway, thus inhibiting the proliferation and migration of tumour cells. Ueki et al. also found that UBE2T plays a key role in the carcinogenesis and progression of breast cancer through interaction with and regulation of the BRCA1-associated cyclic domain protein (BARD1) complex [20]. However, the role of UBE2T in ovarian cancer has not been specifically reported. Based on bioinformatic analysis, Zou et al. reported that UBE2T might play a role in the occurrence and development of ovarian cancer [33]; however, the specific mechanism involved in this process has not been investigated.

In this study, bioinformatic analysis revealed that UBE2T was highly expressed in ovarian cancer, and this high expression was closely related to the poor prognosis of patients. Moreover, it was found that UBE2T may influence the occurrence and development of ovarian cancer by affecting tumour cell proliferation and invasion, intercellular adhesion, and extracellular matrix decomposition and synthesis. Subsequently, immunohistochemistry was used to verify the high expression of UBE2T in ovarian cancer. The analysis demonstrated that the expression levels of UBE2T were higher in ovarian cancer cells with a *BRCA* gene mutation. These results suggested that UBE2T may be expressed as an oncogene in the occurrence and development of ovarian cancer, potentially promoting its occurrence, development and metastasis. *BRCA* gene mutations are the main cause of malignant alterations in patients with epithelial ovarian cancer. Approximately 10%–15% of epithelial ovarian malignant tumours are caused by germline mutations of the *BRCA* gene, and 20%–25% of high-grade serous ovarian cancers occur in patients with germline *BRCA* mutations [34–36]. BRCA-mediated DNA repair is important for maintaining normal differentiation and inhibiting the development of breast and ovarian cancer [37, 38]. The present study was the first to demonstrate that the expression of UBE2T is higher in ovarian cancer cells with a *BRCA* gene mutation. This finding shows that there is a significant correlation between the *UBE2T* and *BRCA* genes, suggesting that interaction between these genes may affect the occurrence and development of ovarian cancer.

At present, increasing evidence shows that EMT can induce metastasis, ascites formation and resistance to chemotherapy in ovarian cancer [14]. Therefore, it may be possible to effectively treat ovarian cancer and delay the progression of the disease by inhibiting EMT. EMT is a reversible cellular process in which epithelial cells are temporarily in a quasi-mesenchymal state [13, 39]. The malignant progression of numerous types of cancer may depend entirely on the activation of EMT in tumour cells [40–42]. During tumour development, EMT endows a single cancer cell with various characteristics associated with high-grade malignant tumours [41–43]. In cancer, EMT enables cancer cells with epithelial phenotypes to acquire stromal characteristics [44]. EMT also plays an important role in the malignant development of tumours, including tumour initiation, migration and invasion of tumour mesenchymal cells, resistance to apoptosis, immune escape, resistance to chemotherapy, malignant progression, differentiation of tumour stem cells, tumour cell migration, vascular infiltration and metastasis [13, 45, 46]. This study found that the proliferation and invasion of ovarian cancer cells were significantly inhibited after UBE2T silencing. Ovarian cancer cells showed increased expression of E-cadherin and decreased expression of V-cadherin and vimentin. These findings indicated that ovarian cancer cells transformed from a stromal cell phenotype to an epithelial cell phenotype, revealing that EMT of ovarian cancer cells was significantly inhibited. This suggests that UBE2T may promote the proliferation, invasion and metastasis of ovarian cancer by promoting EMT in cancerous cells. Numerous studies reported that deletion of the *BRCA* gene is related to EMT and tumorigenesis. The link between *BRCA* and EMT in breast cancer has been previously demonstrated [47, 48]; nevertheless, thus far, this relationship has not been studied in ovarian cancer. Of note, decreased expression of *BRCA* can induce the number of tumour-initiating cells, EMT and stem cells in breast cancer [49]. Various associations between key molecules of BRCA and EMT have been found, which may explain the frequent development of highly invasive and poorly differentiated serous ovarian cancer in patients with *BRCA* mutations. However, this hypothesis and the specific mechanism involved in the relationship between *BRCA* and EMT warrant further investigation. This study found a significant correlation between UBE2T and BRCA. UBE2T may promote the progression of ovarian cancer by promoting the occurrence of EMT in ovarian cancer cells. This indicates that the UBE2T gene may be the key link in the interaction between BRCA and EMT. At present, patients with BRCA mutations are effectively treated with poly(ADP-ribose) polymerase (PARP) inhibitors. Nevertheless, future research on UBE2T and BRCA may offer more beneficial treatment options to patients with ovarian cancer.

Following specific signals released by cells that form the matrix microenvironment, several intracellular signal pathways (eg PI3K-AKT pathway) are activated in epithelial cells. This effect promotes cell growth and proliferation by inducing the occurrence of EMT and enhances cell migration and movement [50–52]. The PI3K-AKT pathway is activated in 40% of ovarian cancers, and this activation is associated with poor prognosis [53, 54].

In this study, we found that the PI3K-AKT pathway and the proliferative and invasive ability of ovarian cancer cells were significantly inhibited following UBE2T silencing in ovarian cancer cell lines. Treatment with the mTOR activator MHY1485 activated the PI3K-AKT pathway and significantly restored the proliferative and invasive ability of ovarian cancer cells. These findings suggest that the PI3K-AKT pathway is the key signalling pathway involved in the regulation of the proliferation and invasion of ovarian cancer cells by UBE2T. UBE2T may regulate the EMT process of ovarian cancer cells through mTOR targets in PI3K-AKT pathway.

In addition, we also observed that the growth of ovarian cancer tumours on the body surface of mice was significantly inhibited after the silencing of UBE2T in SCID mice. Analysis of the expression of E-cadherin, N-cadherin and vimentin in tumour tissue, demonstrated that EMT in tumour tissue was significantly inhibited following UBE2T silencing. Importantly, these observations were consistent with the results of previous *in vitro* experiments in ovarian cancer cells. Taken together, the findings suggested that UBE2T promotes the occurrence and development of ovarian cancer by promoting EMT in cancerous cells.

## Conclusions

In this study, we found that the expression of UBE2T was significantly increased in ovarian cancer cell lines and tissues. Moreover, the downregulation of UBE2T expression could inhibit the proliferation of ovarian cancer cells. The expression of UBE2T is significantly increased in ovarian cancer cells with a BRCA mutation. This suggests an interaction between the UBE2T and BRCA genes, which affects the occurrence and development of ovarian cancer. Further investigation showed that knockout of the UBE2T gene can inhibit the expression of genes downstream of the PI3K-AKT pathway. Also, UBE2T may promote the proliferation and invasion of ovarian cancer cells through the PI3K-AKT pathway. Following inhibition of UBE2T, the surface phenotype of ovarian cancer cells changes from the stromal phenotype to the epithelial phenotype, indicating that EMT is inhibited in ovarian cancer cells. Therefore, UBE2T may promote the occurrence and development of ovarian cancer by promoting EMT. This study shows that UBE2T plays a carcinogenic role in ovarian cancer by regulating EMT through the PI3K-AKT pathway. This investigation was the first to show a significant correlation between UBE2T and *BRCA* mutation and that UBE2T can promote EMT occurrence. Although the relationship between BRCA and EMT has been confirmed by numerous studies, we will continue to investigate whether UBE2T and BRCA can promote the occurrence of EMT. The present findings suggest that UBE2T may be a valuable new marker for the early diagnosis and prognosis of ovarian cancer. Furthermore, the inhibition of UBE2T may be a new target for the treatment of ovarian cancer.

## Methods

### Bioinformatic analysis

We retrieved and downloaded ovarian cancer data from the Gene Expression Omnibus database: GSE51088. The characteristics of patients in this dataset is shown below. Ovarian tissues and matched peripheral blood samples were prospectively obtained from sequential patients undergoing planned gynecologic surgery at Cedars-Sinai Medical Center between 1989 and 2005. All patients underwent surgery and received adjuvant chemotherapy with a contemporaneous standard-of-care regimen. The bioinformatic analysis was performed using the R software. The differentially expressed genes were analysed by fold change and *t*-test. The thresholds of adjusted *P*-values < 0.05 and |log2foldchange|> 2 were used to screen differentially expressed genes. Spearman rank correlation analysis was used to analyse the correlation between UBE2T and BRCA1&2. The R-packet survival was used for survival analysis. Enrichment analysis was carried out using Enrichr (https://maayanlab.cloud/Enrichr/). To explore biological information and obtain more comprehensive data regarding gene and protein functions, we used clusterProfile packages for Gene Ontology (GO) (including cellular component, biological process and molecular function) and Kyoto Encyclopedia of Genes and Genomes (KEGG) analyses [55].

### Tissues and cell lines

Ovarian cancer samples and normal ovary tissues were collected from 125 patients who underwent surgery at Harbin Medical University Cancer Hospital (Harbin, China) between December 2018 and December 2020. Patients with ovarian cancer had not received radiotherapy or chemotherapy prior to surgery. Written informed consent for the use of the tissue samples for research purposes was provided by all patients. All procedures were conducted following the principles outlined in the Declaration of Helsinki. The study protocol was approved by the Ethics Committee of Harbin Medical University (Harbin, China).

The ovarian cancer cell lines (CAOV3, SKOV3, ES2, HO8910 and A2780) were obtained from the Cell Bank of Chinese Academy of Sciences (Shanghai, China). The normal ovary cell line (IOSE80) was purchased from the American Type Cell Culture Collection (Rockville, MD, USA). Cells were incubated in Dulbecco’s modified Eagle’s medium (DMEM; cat. no. 670087; Gibco; Thermo Fisher Scientific, Inc.) supplemented with 10% fetal bovine serum (FBS; cat. no. 16140071; Gibco; Thermo Fisher Scientific, Inc.) and maintained at 37 °C in an atmosphere containing 5% CO_2_. The signalling pathway activator MHY1485 (cat. no. S7811) and mitochonic acid 5 (cat. no. S0881) were purchased from SelleckChem. The mTOR activator (MHY1485) was purchased from MCE (Shanghai, China).

### Immunohistochemistry

Immunohistochemistry was performed using UBE2T antibody (cat. no. 10105–2-AP; Proteintech Group, Inc) and biotinylated anti-rabbit secondary antibody (cat. no. PV-6001; ZSGB-BIO, Inc). In brief, the sections were subjected to deparaffinisation and rehydration. Following antigen retrieval, tissue sections were incubated with UBE2T antibody (cat. no. 10105–2-AP; Proteintech Group, Inc) overnight at 4 °C. After washing with phosphate-buffered saline, sections were incubated with biotinylated anti-rabbit secondary antibody (cat. no. PV-6001; ZSGB-BIO, Inc) for 1 h at room temperature. The diaminobenzidine kit was used as a chromogen, and the slides were counterstained with hematoxylin. The immunohistochemically stained tissue sections were independently scored by two pathologists blinded to the clinical parameters. The staining intensity was scored as follows: 0 (negative), 1 (weak), 2 (medium) and 3 (strong). The extent of staining was scored as 1 (1%–25%), 2 (26%–50%), 3 (51%–75%) and 4 (76%–100%), according to the percentages of the positive staining areas compared to the entire carcinoma-involved area or the entire section for normal samples. The sum of the intensity and extent scores was regarded as the final staining score (0–12) for UBE2T. For statistical evaluation, a final staining score of ≥ 3 in tumours denoted high UBE2T expression.

### Reverse transcription quantitative polymerase chain reaction (RT-qPCR)

Total RNA was collected from tumour cells using TRIzol Reagent (cat. no. R1021; Beijing Transgen Biotech Co., Ltd., Beijing, China) and reversely transcribed into cDNA using the PrimeScript™ RT Kit (cat. no. RR014A; Takara Biotechnology Co., Ltd.) according to the instructions provided by the manufacturers. RT-qPCR was conducted thrice on an ABI 7500HT Real-Time PCR instrument (Applied Biosystems; Thermo Fisher Scientific, Inc.) according to the instructions provided by the manufacturer. The thermocycling conditions were as follows: initial denaturing step (94 °C, 10 min), followed by 40 cycles of denaturing (94 °C, 5 s), annealing (60 °C, 30 s) and extending (72 °C, 45 s). The primers sequences used are presented in Table 3. To verify the expression of UBE2T, endogenous mRNA was synthesised using a SYBR Green PCR Master Mix Kit (cat. no. 4309155; Invitrogen; Thermo Fisher Scientific, Inc.).

**Table 3:** Primers for RT-qPCR for UBE2T and GAPDH

Gene	Forward primer (5'-3')	Reverse primer (5'-3')
UBE2T	CGAGCTCGTAGAAATATTAGGTGGA	TCATCAGGGTTGGGTTCTGA
GAPDH	AAGAAGGTGGTGAAGCAGGC	GTCAAAGGTGGAGGAGTGGG

*UBE2T* Ubiquitin-conjugating enzyme E2T, *GAPDH* Glyceraldehyde-3-phosphate dehydrogenase

### Western blotting

Total protein was extracted from cell lines (SKOV3 and HO8910) and mouse tissue samples (250 mg/sample) using radio immunoprecipitation assay lysis buffer (cat. no. P0013D; Beyotime Institute of Biotechnology, Shanghai, China) containing a protease inhibitor cocktail (Complete™ Mini; Roche Applied Science) at 4 °C for 10 min. Protein concentration was measured using a bicinchoninic acid protein assay kit (cat. no. P0012S; Beyotime Institute of Biotechnology). Proteins (40 µg) were separated by 10% sodium dodecyl sulfate–polyacrylamide gel electrophoresis and transferred onto polyvinylidene difluoride membranes (Beyotime Institute of Biotechnology). Membranes were blocked with tris-buffered saline containing 5% non-fat milk (weight/volume) for 1 h at room temperature and incubated with primary antibodies overnight at 4 °C. Subsequently, the membranes were incubated with horseradish peroxidase-conjugated anti-mouse or anti-rabbit (cat. no. ab6721 and ab6728; 1:2,000; Abcam) secondary antibodies for 1 h at room temperature. The bands were visualised through enhanced chemiluminescence detection (Beyotime Institute of Biotechnology) with a ChemiDoc™ MP Imaging System and analysed using the Image Lab software (version 3.0; Bio-Rad Laboratories, Inc.).

### Plasmids, oligonucleotides and cell transfection

UBE2T small interference RNA (siRNA) and negative control-siRNA (si-NC) were obtained from Guangzhou Ribobio Co., Ltd. (Guangzhou, China). Tumour cells were seeded at the density of 5 × 10^5^ cells per well in six-well plates and cultured in DMEM containing 10% FBS at 37 °C. When the cells reached 70%–80% confluence, they were transfected with 20 μM of each construct using Lipofectamine® 2000 (cat. no. 11668030; Invitrogen; Thermo Fisher Scientific, Inc.) according to the instructions provided by the manufacturer and maintained at 37 °C for 6 h. Next, the culture medium was replaced by fresh DMEM containing 10% FBS, and subsequent experiments were conducted at 24 h post transfection. The construct sequences are shown in Table 4.

**Table 4:** siRNA sequences

si-UBE2T-1	GCAACTGTGTTGACCTCTATT
si-UBE2T-2	TGAGGAAGAGATGCTTGATAA
si-UBE2T-3	GCAACTGTGTTGACCTCTATT
si-NC	GGUAAGCAGUGGCUCCUCUAA

*siRNA* small interfering RNA

### Cell viability assay MTT

The viability of ovarian cancer cells (SKOV3 and HO8910) was evaluated using a MTT Cell Proliferation Kit (cat. no. C0009S; Beyotime Institute of Biotechnology). Tumour cells were seeded in 96-well plates (100 μl containing 3 × 10^3^ cells per well). Each cell line was transfected with si-UBE2T and si-NC. Next, MTT reagent (20 μl) was added to each well, and the cells were incubated at 37 °C for 4 h. The optical density was detected at 450 nm using a Tecan microplate reader (Infinite F50; Tecan Group, Ltd.). All experiments were performed in triplicate.

### Transwell assays

Transfected SKOV3 and HO8910 cells (5 × 10^4^) were seeded into the upper chamber of a Transwell on a Matrigel-coated membrane (cat. no. 3495; Costar; Corning, Inc.) and cultured in serum-free medium for 24 h. DMEM containing 20% FBS was placed in the lower chamber. Subsequently, the medium in the upper chamber was changed, and the cells on the upper side of the filter were removed. The cells that had invaded the lower chamber were fixed with 4% paraformaldehyde at room temperature for 10 min and stained with 0.1% crystal violet (cat. no. IC0600; Beijing Solarbio Science & Technology Co., Ltd., Beijing, China) at room temperature for 10 min. Stained cells were counted in five randomly selected fields under a light microscope (Nikon Corporation) at × 100 magnification.

### *In vivo* xenograft tumour models

Four-week-old, female, severe combined immunodeficiency (SCID) mice (weighing 16–18 g) were obtained from Vital River Laboratory Animal Technology (Beijing, China) and housed under standard conditions. The SKOV3 cells were stably transfected with si-UBE2T or si-NC. Subsequently, cells (5 × 10^5^ cells per mouse in 200 μl) were injected into the axilla of the right forelimb of mice (*n* = 5 per group). After 3 weeks, changes in the weight of each mouse were recorded weekly. The mice were sacrificed using CO_2_ (35% volume displacement per min), and the tumours were excised. Tumour tissues were stored in 4% paraformaldehyde and histologically analysed via hematoxylin–eosin staining. All experiments were performed according to the applicable international, national and/or institutional guidelines for the care and use of animals.

### Statistical analysis

Data were expressed as the means ± standard deviation. Statistical analysis was conducted using the SPSS version 20.0 (IBM Corporation, Armonk, NY, USA) software. One-way analysis of variance with Tukey’s post hoc, chi-squared and Student’s t-tests were used to determine the level of significance between groups. The Kaplan–Meier method and the log-rank test were used for survival analyses using the GraphPad Prism v5.0 (GraphPad Software, Inc., San Diego, CA, USA) software. All experiments were independently performed thrice. *P*-values < 0.05 denoted statistically significant differences.

## Supplementary Information

**Additional file 1: Supplementary Table 1. **The clinical features of patients.

## Data Availability

Contact author for data requests.

## Abbreviations

DMEM: Dulbecco’s modified Eagle’s medium
FBS: Fetal bovine serum
GO: Gene Ontology
KEGG: Kyoto Encyclopedia of Genes and Genomes
MTOR: Mechanistic target of rapamycin
PI3K-AKT: Phosphatidylinositol 3 kinase/protein kinase B
UBE2T: Ubiquitin-binding enzyme E2T
EMT: Epithelial–mesenchymal transition
