# Supplementary material for: UBE2T regulates epithelial–mesenchymal transition through the PI3K-AKT pathway and plays a carcinogenic role in ovarian cancer
Source: J Ovarian Res. 2022 Sep 10;15:103. doi: 10.1186/s13048-022-01034-9 (PMC9464398; doi:10.1186/s13048-022-01034-9)
Supplement: Supplementary file 1 — Additional file 1: Supplementary Table 1. The clinical features of patients. [file 13048_2022_1034_MOESM1_ESM.docx]

**Supplementary table 1** The clinical features of patients

| Group | Admission No. | Pathological Section No. | Age | Dscites | Lymph Node Metastasis | FIGO stage | CA125（g/L） | BRCA |
| --- | --- | --- | --- | --- | --- | --- | --- | --- |
| OC | 804250 | 2006492 | 66 | yes | yes | III | 450.6 | BRCAmut |
| OC | 810128 | 2002224 | 50 | no | yes | III | 42.66 | BRCAmut |
| OC | 797227 | 2015119 | 51 | yes | no | III | 18.3 | BRCAmut |
| OC | 693712 | 1725443 | 51 | yes | yes | III | 3313 | BRCAmut |
| OC | 820127 | 2011144 | 45 | yes | no | II | 462.7 | BRCAmut |
| OC | 817360 | 2014666 | 63 | yes | no | III | 1384 | BRCAmut |
| OC | 803131 | 2015119 | 54 | yes | no | III | 733.1 | BRCAmut |
| OC | 817768 | 2012875 | 49 | no | no | II | 221.4 | BRCAmut |
| OC | 823815 | 2016595 | 44 | yes | no | III | 980.7 | BRCAmut |
| OC | 815640 | 2009441 | 46 | no | no | II | ＞5000 | BRCAmut |
| OC | 816783 | 2022244 | 58 | no | yes | II | 287.8 | BRCAmut |
| OC | 744536 | 1943271 | 46 | yes | no | III | 2386 | BRCAmut |
| OC | 830547 | 2027015 | 62 | no | no | II | 186.1 | BRCAmut |
| OC | 831921 | 2028175 | 58 | yes | yes | I | 1138 | BRCAmut |
| OC | 810450 | 2098852 | 47 | yes | yes | IV | ＞5000 | BRCAmut |
| OC | 827116 | 2029636 | 45 | yes | no | III | 1728 | BRCAmut |
| OC | 675654 | 1721283 | 69 | no | no | II | 231.5 | BRCAmut |
| OC | 831917 | 2037337 | 42 | yes | yes | II | 498 | BRCAmut |
| OC | 834927 | 2038649 | 55 | yes | no | III | 2880 | BRCAmut |
| OC | 839790 | 2038559 | 48 | yes | no | II | 418 | BRCAmut |
| OC | 776529 | 1929743 | 59 | no | no | III | 372 | BRCAmut |
| OC | 709824 | 1743549 | 52 | yes | yes | III | 2814 | BRCAmut |
| OC | 803397 | 1950709 | 48 | no | no | II | 238.3 | BRCAmut |
| OC | 767274 | 1909100 | 48 | yes | no | III | 1025 | BRCAmut |
| OC | 765881 | 1905315 | 37 | no | no | I | 52.58 | BRCAmut |
| OC | 784028 | 1940590 | 46 | yes | yes | III | 1572.4 | BRCAmut |
| OC | 713398 | 1807147 | 67 | no | yes | III | 266.5 | BRCAmut |
| OC | 578143 | 1435595 | 48 | no | no | II | 60 | BRCAmut |
| OC | 808321 | 2020377 | 64 | yes | no | III | 1190 | BRCAmut |
| OC | 749668 | 1837361 | 46 | yes | yes | III | 1298 | BRCAmut |
| OC | 830583 | 2028344 | 50 | no | no | II | 72.58 | BRCAmut |
| OC | 814692 | 2015045 | 60 | no | no | III | 1078 | BRCAmut |
| OC | 772426 | 2008611 | 49 | no | yes | III | 207.1 | BRCAmut |
| OC | 784721 | 1928564 | 46 | yes | yes | III | 3006 | BRCAmut |
| OC | 821439 | 2034036 | 68 | no | no | III | 796.2 | BRCAmut |
| OC | 807623 | 1956264 | 56 | yes | yes | IV | 3812 | BRCAwt |
| OC | 675123 | 1724602 | 60 | no | no | II | 19.43 | BRCAwt |
| OC | 739315 | 1828335 | 61 | no | yes | III | 65 | BRCAwt |
| OC | 813351 | 2006362 | 54 | yes | no | II | 854.5 | BRCAwt |
| OC | 805866 | 2006718 | 52 | yes | no | III | 196 | BRCAwt |
| OC | 809528 | 2008084 | 52 | yes | yes | III | 1204 | BRCAwt |
| OC | 814851 | 2009353 | 66 | no | yes | III | 1118 | BRCAwt |
| OC | 808687 | 2009146 | 47 | yes | no | II | 174 | BRCAwt |
| OC | 819077 | 2011702 | 48 | yes | no | III | 268 | BRCAwt |
| OC | 814565 | 2011223 | 59 | yes | yes | III | 62 | BRCAwt |
| OC | 821570 | 2015599 | 46 | yes | no | III | 434 | BRCAwt |
| OC | 820771 | 2015500 | 56 | yes | no | III | 3293 | BRCAwt |
| OC | 819035 | 2012312 | 43 | yes | no | III | 320 | BRCAwt |
| OC | 820187 | 2011181 | 49 | yes | no | II | 78 | BRCAwt |
| OC | 810464 | 1721283 | 62 | yes | yes | III | 80 | BRCAwt |
| OC | 815762 | 2006263 | 41 | yes | no | II | 39 | BRCAwt |
| OC | 828115 | 2023504 | 57 | no | no | I | 89 | BRCAwt |
| OC | 826383 | 2021403 | 49 | no | no | II | 115 | BRCAwt |
| OC | 826945 | 2021252 | 44 | yes | no | II | 999 | BRCAwt |
| OC | 826266 | 2020846 | 63 | yes | no | II | 1144 | BRCAwt |
| OC | 828593 | 2027326 | 50 | yes | no | II | 69 | BRCAwt |
| OC | 832747 | 2031996 | 64 | yes | no | I | 2199 | BRCAwt |
| OC | 830150 | 2032759 | 51 | yes | yes | III | 672 | BRCAwt |
| OC | 742869 | 1832272 | 52 | yes | yes | III | 2997 | BRCAwt |
| OC | 841484 | 2038828 | 68 | yes | no | III | 1230 | BRCAwt |
| OC | 801029 | 1946495 | 42 | yes | yes | III | 1574 | BRCAwt |
| OC | 737065 | 1825641 | 61 | yes | no | II | 34 | BRCAwt |
| OC | 770226 | 1909267 | 53 | yes | no | III | 176 | BRCAwt |
| OC | 684680 | 1715604 | 54 | no | no | II | 74.9 | BRCAwt |
| OC | 800333 | 1947764 | 62 | no | no | III | 2998 | BRCAwt |
| OC | 800328 | 1947771 | 65 | no | no | III | 93 | BRCAwt |
| OC | 702859 | 1734248 | 57 | yes | no | III | 2568 | BRCAwt |
| OC | 785191 | 1930140 | 52 | yes | no | III | 4972 | BRCAwt |
| OC | 819113 | 2012706 | 50 | yes | no | III | 2478 | BRCAwt |
| OC | 784595 | 1929895 | 51 | no | no | III | 2014 | BRCAwt |
| NC | 841546 | 2039555 | 48 |  |  |  |  |  |
| NC | 825242 | 2039664 | 59 |  |  |  |  |  |
| NC | 839448 | 2039806 | 49 |  |  |  |  |  |
| NC | 832067 | 2036585 | 51 |  |  |  |  |  |
| NC | 837606 | 2039761 | 48 |  |  |  |  |  |
| NC | 837819 | 2036590 | 55 |  |  |  |  |  |
| NC | 842727 | 2039807 | 54 |  |  |  |  |  |
| NC | 841584 | 2039679 | 58 |  |  |  |  |  |
| NC | 837689 | 2039556 | 56 |  |  |  |  |  |
| NC | 839735 | 2039049 | 47 |  |  |  |  |  |
| NC | 833098 | 2039123 | 49 |  |  |  |  |  |
| NC | 839503 | 2039196 | 52 |  |  |  |  |  |
| NC | 839467 | 2038852 | 51 |  |  |  |  |  |
| NC | 825155 | 2038887 | 48 |  |  |  |  |  |
| NC | 839969 | 2038705 | 52 |  |  |  |  |  |
| NC | 841540 | 2038569 | 74 |  |  |  |  |  |
| NC | 839741 | 2038224 | 54 |  |  |  |  |  |
| NC | 838858 | 2038311 | 65 |  |  |  |  |  |
| NC | 839715 | 2038073 | 68 |  |  |  |  |  |
| NC | 841531 | 2038107 | 58 |  |  |  |  |  |
| NC | 832199 | 2037336 | 55 |  |  |  |  |  |
| NC | 832147 | 2037074 | 62 |  |  |  |  |  |
| NC | 837857 | 2036977 | 51 |  |  |  |  |  |
| NC | 834600 | 2037276 | 54 |  |  |  |  |  |
| NC | 839746 | 2036697 | 58 |  |  |  |  |  |
| NC | 839540 | 2036550 | 52 |  |  |  |  |  |
| NC | 832120 | 2036534 | 64 |  |  |  |  |  |
| NC | 832081 | 2036728 | 65 |  |  |  |  |  |
| NC | 821331 | 2036264 | 78 |  |  |  |  |  |
| NC | 821296 | 2036283 | 63 |  |  |  |  |  |
| NC | 830188 | 2036274 | 70 |  |  |  |  |  |
| NC | 839089 | 2036044 | 51 |  |  |  |  |  |
| NC | 839070 | 2035230 | 66 |  |  |  |  |  |
| NC | 821268 | 2035313 | 51 |  |  |  |  |  |
| NC | 839221 | 2034977 | 57 |  |  |  |  |  |
| NC | 830189 | 2034879 | 58 |  |  |  |  |  |
| NC | 834946 | 2033921 | 60 |  |  |  |  |  |
| NC | 835039 | 2033642 | 52 |  |  |  |  |  |
| NC | 786598 | 2033664 | 53 |  |  |  |  |  |
| NC | 830178 | 2033589 | 52 |  |  |  |  |  |
| NC | 835185 | 2032987 | 54 |  |  |  |  |  |
| NC | 835400 | 2032355 | 50 |  |  |  |  |  |
| NC | 830146 | 2031324 | 64 |  |  |  |  |  |
| NC | 835308 | 2031620 | 51 |  |  |  |  |  |
| NC | 830008 | 2025275 | 52 |  |  |  |  |  |
| NC | 829866 | 2030789 | 65 |  |  |  |  |  |
| NC | 812042 | 2030954 | 51 |  |  |  |  |  |
| NC | 832796 | 2031153 | 55 |  |  |  |  |  |
| NC | 831856 | 2030117 | 52 |  |  |  |  |  |
| NC | 832768 | 2030784 | 73 |  |  |  |  |  |
| NC | 835294 | 2030526 | 58 |  |  |  |  |  |
| NC | 832967 | 2029540 | 54 |  |  |  |  |  |
| NC | 832907 | 2029510 | 65 |  |  |  |  |  |
| NC | 831908 | 2028720 | 56 |  |  |  |  |  |
| NC | 829965 | 2027918 | 67 |  |  |  |  |  |

OC Ovarian Cancer NC Normal ovarian tissue
